# Enhancers not required

**DOI:** 10.7554/eLife.28339

**Published:** 2017-06-09

**Authors:** Ying Zheng, David Levens

**Affiliations:** Laboratory of Pathology, National Cancer Institute, Bethesda, United States; Laboratory of Pathology, National Cancer Institute, Bethesda, United Stateslevensd@mail.nih.gov

**Keywords:** cancer, oncogene regulation, enhancer, Human, Mouse

## Abstract

Laboratory mice with over half a megabase of DNA upstream of their *Myc* gene removed still thrive in the absence of stress.

**Related research article** Dave K, Sur I, Yan J, Zhang J, Kaasinen E, Zhong F, Blaas L, Li X, Kharazi S, Gustafsson C, De Paepe A, Månsson R, Taipale J. 2017. Mice deficient of *Myc* super-enhancer region reveal differential control mechanism between normal and pathological growth. *eLife* **6**:e23382. doi: 10.7554/eLife.23382

The transcription of a gene starts with gene-regulating proteins binding to a nearby region of DNA called a promoter, and can be up-regulated when other proteins bind to other more distant regions of DNA called enhancers (Khoury and Gruss, 1983). Super-enhancers are DNA sequences that are studded with typical enhancers and other regulatory elements. They strongly activate genes and may span tens of thousands of DNA bases (Hnisz et al., 2013). Because DNA molecules have to be folded to fit inside the nucleus, enhancers and super-enhancers can come into contact with many genes and act across vast distances. Indeed, it is possible for an enhancer or super-enhancer to act on a gene that is separated from it by more than a megabase (that is, there may be over one million bases between the gene and the enhancer or super-enhancer).

*MYC* is a regulatory gene that is usually made at low levels; high output is virtually always short-lived in normal cells. The *MYC* gene is also a proto-oncogene: this means that it can trigger cancer if it is continuously overexpressed, which happens in most cancers (Levens, 2013). The region around *MYC* is largely free from other genes, and mutations that bring strong enhancers or super-enhancers into this gene-free region drive the expression of *MYC* in many tumors. The high levels of *MYC* expression in other tumors are sustained by smaller changes that bring gene-activating proteins to the enhancers that are already found on either side of the gene. However, it was not known how the inputs of these enhancers are integrated in living animals. It was also not clear if the many enhancers found around *MYC* ever coalesce to form a super-enhancer.

Now, in eLife, Jussi Taipale and colleagues – including Kashyap Dave and Inderpreet Sur as joint first authors – report on the role of the enhancer regions around the equivalent gene in mice, which is referred to as *Myc* (Dave et al., 2017). Previously, this group had removed a likely enhancer far upstream of *Myc *(Sur et al., 2012). Other than having slighty less Myc protein in the colon, these mice were normal, and in fact were more resistant to colon cancer. Dave et al. – who are based at the Karolinska Institute and University of Helsinki – expected that deleting a much larger upstream region would cripple *Myc* expression and have a much more harmful effect. Instead, they found that mice that lack over half a megabase of DNA upstream of their *Myc* gene still develop as normal and are fertile. Notably, Dave et al. refer to this large region as a “super-enhancer region”, but the justification for this moniker is somewhat tenuous at present (Pott and Lieb, 2015).

Further analysis revealed that without the enhancer region the background levels of *Myc* gene expression is lower in some tissues, but higher in the spleen. Nevertheless, the major effect seen in the mice (which are referred to as “enhancer-minus mice”) seems to be greater resistance to tumors. When challenged by a carcinogen or the loss of a tumor suppressor gene, the enhancer-minus mice are less susceptible to developing tumors in their mammary glands and intestines (two tissues that have lower *Myc* levels when the enhancer region is deleted).

Why can most tissues seemingly carry on as normal regardless of the level of Myc? When abundant, the Myc protein binds to and amplifies the expression of almost every active gene (Levens, 2013; Wolf et al., 2015). This means that this one protein can influence how a cell responds to a myriad of signals. However, Dave et al. show that even when *Myc* expression in the colon drops by 80%, the expression of most other genes seems largely unchanged. Skin cells (specifically fibroblasts) in enhancer-minus mice also seem indistinguishable from their wild-type counterparts, but they do proliferate more slowly when grown in the laboratory.

Dave et al. try to reconcile the paradox of Myc being important in tissue culture and tumors, but seeming to be irrelevant in actual animals with an “off-on” model. Their model defines the low levels of Myc in the colon as “off”, and the Myc in cells exposed to the growth-promoting signals used in tissue culture as “on”. However, it is hard to believe that the Myc in the colon is really “off” and not functional. Though unappreciated at the level of single genes, taking a closer look at their data reveals that the most highly expressed genes in the colons of enhancer-minus mice are noticeably expressed less than those in wild-type mice (Figure 1). In contrast, the increased Myc in the spleen boosts the expression of genes that were previously expressed at intermediate levels. So it seems that it is not that Myc is incapacitated at reduced levels: rather, cells are robust enough to resist some fluctuations in the level of Myc.

**Figure 1.**
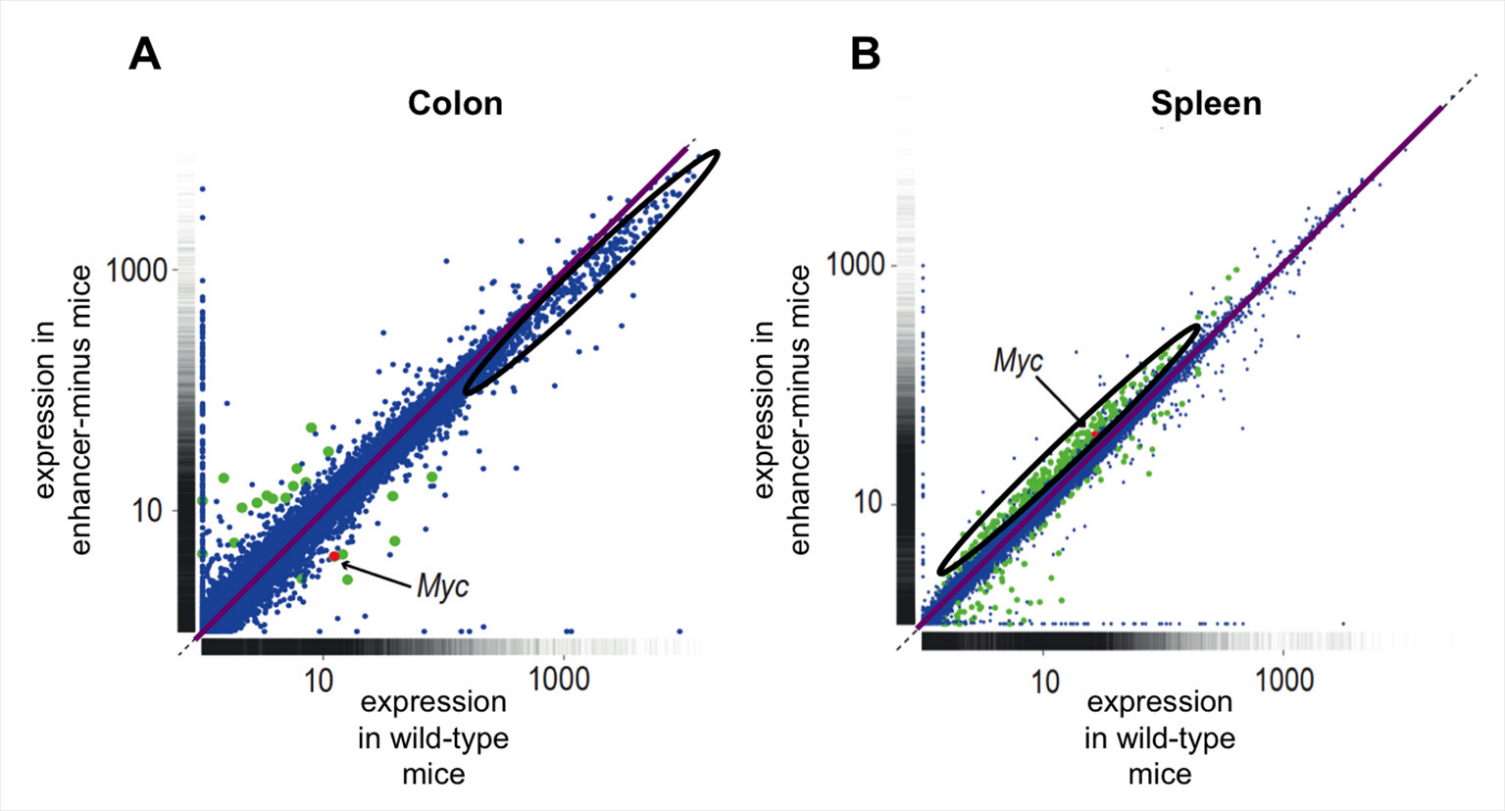
Comparing gene expression in mouse cells with and without the enhancer region upstream of *Myc*. Each dot represents a gene: the *Myc* gene is marked in red; genes that show significantly different expression between the two types of mice are marked in green; all other genes are marked in blue. The diagonal purple lines indicate where the expression in enhancer-minus mice is equal to that in wild-type mice. Dots above the purple line are expressed more in the enhancer-minus mice, while dots below the line are expressed less in these mice. (**A**) Gene expression in colon cells remains largely unchanged between wild-type and enhancer-minus mice. However, the oval region delineates genes at the high end of the expression spectrum that depend upon *Myc* for full expression in wild-type mice; these genes are expressed less highly in the enhancer-minus mice because *Myc* levels are depressed in the colon (However, this difference is not significant at the level of individual genes). (**B**) Gene expression in spleen cells is different between the two types of mice. The oval region delineates genes with intermediate levels of expression that are increased due to the higher *Myc* levels in the spleens of the enhancer-minus mice. Figure adapted from Figure 5 of Dave et al. (2017).

The different effects of Myc on target genes in the colon, cultured fibroblasts, and in the spleen can be explained by an existing model. This model proposes that the strength of Myc binding with a gene’s promoter generally parallels the expression levels of the gene and that Myc will saturate the highest affinity promoters before binding to the weaker ones (Lorenzin et al., 2016). Myc levels can be ordered as: lowest in the colon, intermediate in the spleen, and highest in cultured fibroblasts. With lower levels in the colon, there is insufficient Myc to bind to all but the most highly expressed genes – and it is exactly these genes that deviate downward in the colon as the levels of Myc diminish further when the enhancer region is deleted (Figure 1A). The higher *Myc* levels in the spleens of enhancer-minus mice saturate the most expressed and highest affinity promoters and the excess spills over to act upon the previously unamplified gene targets (Figure 1B).

So, the background level of Myc in a tissue defines which genes will respond if Myc levels increase or decrease. Even when Myc activity is too low to sustain a biologically meaningful response, as in the colon, boosting its background levels may make the cells more likely to deploy responses that are already ready and waiting within a cell should they receive the correct signal (Wolf et al., 2015; Nie et al., 2012). In the absence of stress, animals with reduced Myc levels thrive and outlive their wild-type brethren. By raising Myc levels and increasing the flux through a host of signaling pathways, a cell may better manage physiological stresses and endure threats that may otherwise cause disease.

## References

[bib1] Dave K, Sur I, Yan J, Zhang J, Kaasinen E, Zhong F, Blaas L, Li X, Kharazi S, Gustafsson C, De Paepe A, Månsson R, Taipale J (2017). Mice deficient of *Myc* super-enhancer region reveal differential control mechanism between normal and pathological growth. eLife.

[bib2] Hnisz D, Abraham BJ, Lee TI, Lau A, Saint-André V, Sigova AA, Hoke HA, Young RA (2013). Super-enhancers in the control of cell identity and disease. Cell.

[bib3] Khoury G, Gruss P (1983). Enhancer elements. Cell.

[bib4] Levens D (2013). Cellular MYCro economics: Balancing MYC function with *MYC* expression. Cold Spring Harbor Perspectives in Medicine.

[bib5] Lorenzin F, Benary U, Baluapuri A, Walz S, Jung LA, von Eyss B, Kisker C, Wolf J, Eilers M, Wolf E (2016). Different promoter affinities account for specificity in MYC-dependent gene regulation. eLife.

[bib6] Nie Z, Hu G, Wei G, Cui K, Yamane A, Resch W, Wang R, Green DR, Tessarollo L, Casellas R, Zhao K, Levens D (2012). c-Myc is a universal amplifier of expressed genes in lymphocytes and embryonic stem cells. Cell.

[bib7] Pott S, Lieb JD (2015). What are super-enhancers?. Nature Genetics.

[bib8] Sur IK, Hallikas O, Vähärautio A, Yan J, Turunen M, Enge M, Taipale M, Karhu A, Aaltonen LA, Taipale J (2012). Mice lacking a *Myc* enhancer that includes human SNP rs6983267 are resistant to intestinal tumors. Science.

[bib9] Wolf E, Lin CY, Eilers M, Levens DL (2015). Taming of the beast: shaping Myc-dependent amplification. Trends in Cell Biology.

